# Hesperidin identified from *Citrus* extracts potently inhibits HCV genotype 3a NS3 protease

**DOI:** 10.1186/s12906-022-03578-1

**Published:** 2022-04-02

**Authors:** Mahim Khan, Waqar Rauf, Fazal-e- Habib, Moazur Rahman, Shoaib Iqbal, Aamir Shehzad, Mazhar Iqbal

**Affiliations:** 1 Health Biotechnology Division, Pakistan Institute of Engineering and Applied Sciences, National Institute for Biotechnology and Genetic Engineering College, (NIBGE-C, PIEAS), Faisalabad, Punjab 38000 Pakistan; 2 School of Biological Sciences, University of the Punjab, Lahore, 54810 Punjab Pakistan

**Keywords:** NS3 protease, HCV genotype 3a, FRET assay, *Citrus* plant extract, Hesperidin, Mass spectrometry

## Abstract

**Background:**

Hepatitis C virus infection is the main cause of liver ailments across the globe. Several HCV genotypes have been identified in different parts of the world. Effective drugs for combating HCV infections are available but not affordable, particularly to infected individuals from resource-limited countries. Hence, cost-effective drugs need to be developed against important HCV drug targets. As *Citrus* fruits naturally contain bioactive compounds with antiviral activities, the current study was designed to identify antiviral inhibitors from *Citrus* fruit extracts against an important drug target, NS3 protease, of HCV genotype 3a which is found predominantly in South Asian countries.

**Methods:**

The full-length NS3 protease alone and the NS3 protease domain in fusion with the cognate NS4A cofactor were expressed in *Escherichia coli*, and purified by chromatographic techniques. Using the purified protein as a drug target, *Citrus* extracts were evaluated in a FRET assay, and active ingredients, identified using ESI–MS/MS, were docked to observe the interaction with active site residues of NS3. The best interacting compound was further confirmed through the FRET assay as the inhibitor of NS3 protease.

**Results:**

Fusion of the NS3 protease domain to the NS4A cofactor significantly improved the purification yield, and NS3-NS4A was functionally more active than the full-length NS3 alone. The purified protein (NS3-NS4A) was successfully employed in a validated FRET assay to evaluate 14 *Citrus* fruit extracts, revealing that the mesocarp extract of *Citrus paradisi*, and whole fruit extracts of *C. sinesis*, *C. aurantinum*, and *C. reticulata* significantly inhibited the protease activity of HCV NS3 protease (IC_50_ values of 5.79 ± 1.44 µg/mL, 37.19 ± 5.92 µg/mL, 42.62 ± 6.89 µg/mL, and 57.65 ± 3.81 µg/mL, respectively). Subsequent ESI-MS^n^ analysis identified a flavonoid, hesperidin, abundantly present in all the afore-mentioned *Citrus* extracts. Importantly, docking studies suggested that hesperidin interacts with active site residues, and acts as a potent inhibitor of NS3 protease, exhibiting an IC_50_ value of 11.34 ± 3.83 µg/mL.

**Conclusions:**

A FRET assay was developed using NS3-NS4A protease, which was successfully utilized for the evaluation of *Citrus* fruit extracts. Hesperidin, a compound present in the *Citrus* extracts, was identified as the main flavonoid, which can serve as a cost-effective potent inhibitor of NS3 protease, and could be developed as a drug for antiviral therapy against HCV genotype 3a.

**Supplementary Information:**

The online version contains supplementary material available at 10.1186/s12906-022-03578-1.

## Background

Hepatitis C, caused by hepatitis C virus (HCV), is a leading cause of liver-related health problems. Acute HCV infections are often asymptomatic, whereas chronic HCV infections (accounting for ~ 70% of HCV infections) can lead to the development of clinical symptoms such as liver cirrhosis, hepatocellular carcinoma, and liver failure [1, 2], ultimately leading to the death of infected individuals [3]. It has been estimated that each year ~ 1.5 million people develop chronic HCV infections, resulting in the death of ~ 290,000 infected individuals worldwide [3].

HCV harbors a 10 kb-long RNA genome which displays a high level of genetic variation due to the lack of a proof-reading activity of its RNA-dependent RNA polymerase enzyme, resulting in the emergence of different HCV genotypes and subtypes across the globe [4]. So far, at least six (6) genotypes of HCV have been identified, with multiple subtypes [5]. Different HCV genotypes are prevalent in different parts of the world. Genotypes 1 and 3 are the two most common genotypes of HCV [6, 7]. Genotypes 2, 3, 4, 5, and 6 are found predominantly in West Africa and Americas, the Indian sub-continent and Southeast Asia, Central and East Africa, South Africa, and Southeast Asia, respectively [5].

The HCV genome encodes 3 structural (core and envelope (E1 and E2) proteins) and 7 non-structural proteins (p7, NS2, NS3, NS4A, NS4B, NS5A, and NS5B) [8]. HCV proteins which have so far been targeted for the discovery of direct-acting anti-HCV drugs include NS3-NS4A protease, NS5A, and NS5B polymerase [9, 10]. Recently, NS3 protease has gained enormous attention as an important drug target for the development of direct-acting anti-HCV drugs. NS3 protease is a multifunctional enzyme that can perform a serine protease activity (through its N-terminal domain) and an RNA helicase activity (through its C-terminal domain), and is implicated in viral processing and maturation [11]. For performing the protease activity, an activator (cofactor) peptide, NS4A, is also required. Inhibiting HCV maturation by blocking the activity of NS3 protease is, therefore, a promising therapeutic strategy. It has been shown that direct-acting drugs developed against NS3 protease, or other drug targets, are more effective and less harmful in combating HCV infections than the previous treatment option, which was based on pegylated interferon and ribavirin [9, 10, 12–14]. Recently, a drug (glecaprevir) with a pan-genotype activity against HCV has been identified. Together with an NS5A inhibitor (pibrentasvir), the United States Food and Drug Administration (FDA) has approved glecaprevir for the treatment of HCV. However, a major drawback which limits the widespread use of glecaprevir for the treatment of HCV is its high cost, limiting the affordable access to therapy for a large proportion of infected individuals in resource-limited and developing countries. Furthermore, the number of HCV infections is still increasing despite the availability of effective drugs, making it highly challenging to achieve the World Health Organization’s (WHO) target of hepatitis C elimination by 2030 [3]. Therefore, there is an urgent need to develop highly efficacious and cost-effective drugs for the treatment of HCV, especially for use in resource-limited and developing countries of the world.

Plants are a rich source of natural compounds with diverse pharmaceutical properties [15, 16]. In particular, different parts of plants naturally contain abundant amounts of polyphenols (such as flavonoids), which have demonstrated antimicrobial activities against a wide range of pathogens, including viruses [17–28]. Plant-derived flavonoids have also been shown to possess anti-HCV activities, and could be used as cost-effective alternatives for the treatment of HCV [29–31]. As *Citrus* fruits naturally contain bioactive metabolites, especially flavonoids, which exhibit diverse bioactivities such as anti-oxidative, anti-cancer, anti-inflammatory, and antiviral properties [32], we focused our efforts on the identification of compounds from *Citrus* fruit extracts that can potently inhibit NS3 protease of HCV genotype 3a. For this purpose, extracts from 14 *Citrus* fruits were prepared and evaluated against the purified NS3 protease enzyme, complexed with the NS4A cofactor, of HCV genotype 3a in a FRET assay. *Citrus* extracts exhibiting anti-HCV properties were subsequently analyzed through the ESI–MS/MS technique for the identification of potent compounds, and insights into the molecular bases for the anti-HCV NS3 protease properties of identified compounds were gained through molecular docking studies.

## Methods

### Plasmid constructs

The plasmid (pET11a-His_6_-NS3) (Drug Discovery and Structural Biology Lab, NIBGE, Pakistan) encoding the full-length NS3 protease of HCV genotype 3a fused to an oligohistidine (His_6_) tag at the N-terminus was used for protein expression, as described previously [33]. A synthetic construct (pET11a-His_7_-NS3-NS4A) encoding NS3 protease of HCV genotype 3a covalently linked to the NS4A cofactor (at the C-terminus) and an oligohistidine (His_7_) tag (at the N-terminus) was commercially purchased (GenScript, Piscataway, NJ, United States).

### Heterologous expression of target proteins in *Escherichia coli*

Target proteins (the full-length NS3 and NS3-NS4A) fused to oligohistidine tags were expressed in *Escherichia coli* BL21 (DE3) (Novagen, USA) or *E. coli* BL21-CodonPlus (DE3)-RIL cells (Agilent, USA). For protein expression, an overnight culture of transformed cells was prepared by inoculating 1.5 mL of the LB medium (Sigma Aldrich), supplemented with ampicillin (125 µg/mL) (Sigma Aldrich), with a single transformed colony and incubating the medium overnight at 37 °C with shaking at 225 rpm. Then, 150 mL of the LB medium, supplemented with ampicillin (125 µg/mL), taken in a 1-L flask, was inoculated with the overnight culture, and the flask culture was continued until OD_600_ reached 0.5–0.6. The culture was subsequently incubated at 4 °C for 30 min, and supplemented with 100 µM zinc chloride (Sigma Aldrich). Expression of recombinant proteins was induced by adding 1 mM isopropyl-β-D-thiogalactoside (IPTG) (Thermo Fisher Scientific), and the cells were cultivated for 16 h (at 18 °C) and 4 h (at 30 °C) for the expression of the full-length His_6_-NS3 and His_7_-NS3-NS4A, respectively. The cells were harvested by centrifugation (12,000 × g) at 4 °C for 30 min. After discarding the supernatant, the cell pellet was stored at -80 °C until further use. For the analysis of the protein expression at a small scale, cells were lysed using a bacterial protein extraction reagent (B-PER, Thermo Fisher Scientific), and proteins were analyzed on a 4–12% Bis–Tris NuPAGE gel using an X-Cell Surelock Mini-cell electrophoresis system (Invitrogen).

### Protein purification through affinity chromatography and gel filtration

The cell pellet was resuspended in buffer A (25 mM HEPES (Thermo Fisher Scientific) pH 7.5, 1 M NaCl (Riedel), 10% glycerol (Invitrogen), and 25 mM imidazole (Sigma Aldrich) to prevent non-specific binding of proteins to the column during affinity chromatography), and lysed through a high-pressure homogenizer (pressure: 10 kpsi; 2 passes; APV-1000 homogenizer, Invensys APV Products, Denmark). The cell lysate was centrifuged at 30,000 × g (30 min), and the supernatant was clarified by passing it through a 0.45 µm membrane filter. The clarified cell extract was loaded on a His-Trap column (GE, Healthcare), connected to an NGC system, pre-equilibrated with buffer A. After washing the column with buffer A to the baseline, proteins were eluted by applying a linear imidazole gradient (25 mM to 250 mM for His_6_-NS3, and 25 mM to 500 mM for His_7_-NS3-NS4A). The proteins were dialysed against the dialysis buffer (25 mM HEPES, pH 7.6, 0.2 M NaCl, 20% glycerol, 0.4% Triton X-100 (Thermo Fisher Scientific), and 10 mM β-mercaptoethanol (Roth, Germany)) at 4 °C overnight, and concentrated using an Amicon Ultracentrifugal filter (10 kDa molecular-weight cut-off; EMD Millipore Corporation, Billerica, MA). The His_6_-NS3 protein was subjected to size-exclusion chromatography using a 120 mL HiPrep 16/60 Sephacryl S-200 column (GE Healthcare Bio-Sciences Corporation, Piscataway, NJ), pre-equilibrated with the dialysis buffer. The His_7_-NS3-NS4A protein was diluted using buffer (25 mM HEPES, pH 7.5, 1 M NaCl, 10% glycerol) to decrease the imidazole concentration to 25 mM, and then incubated with His_6_-tagged TEV protease at 4 °C overnight, followed by incubation at the room temperature for 4 h, in order to cleave the oligohistidine tag. The target protein (NS3-NS4A) was separated from the cleaved tag and His_6_-tagged TEV protease through reverse affinity chromatography by applying the digestion mixture on a fresh His-Trap column (GE, Healthcare), pre-equilibrated with buffer A. The flow-through from the column was concentrated using an Amicon Ultracentrifugal filter (10 kDa molecular-weight cut-off; EMD Millipore Corporation, Billerica, MA), and subjected to size-exclusion chromatography using a 120 mL HiPrep 16/60 Sephacryl S-200 column (GE Healthcare Bio-Sciences Corporation, Piscataway, NJ), pre-equilibrated with buffer A. The purified proteins (His_6_-NS3 and NS3-NS4A) were concentrated using Amicon Ultracentrifugal filters (10 kDa molecular-weight cut-off; EMD Millipore Corporation, Billerica, MA), and were either immediately used for the enzymatic assay or flash-frozen in liquid nitrogen and stored at -80 °C until further use. The protein concentration was estimated using extinction coefficients (calculated through the ExPasy ProtParam tool) of 71,500 M^−1^ cm^−1^ and 18,700 M^−1^ cm^−1^ for His_6_-NS3 and NS3-NS4A, respectively.

### Activity measurement of purified proteins

For measuring the protease activity, a FRET assay was performed using a depsipeptide substrate, Ac-Asp-Glu-Asp-(EDANS)-Glu-Glu-Abu-ψ-[COO]-Ala-Ser-Lys(DABCYL)-NH2 (AnaSpec,US). The fluorescence signal generated upon cleavage of the depsipeptide substrate by the NS3 protease domain was continuously recorded using excitation and emission wavelengths of 355 nm and 510 nm, respectively, and a fluorescence microplate reader (TECAN, US). In the case of the full-length NS3 protease, the reaction buffer (50 mM HEPES, pH 7.5, 0.4% Triton X-100, 10 mM DTT (Thermo Fisher Scientific), 3% DMSO (Thermo Fisher Scientific), and 40% glycerol) was pre-incubated (at 30 °C for 10 min) with 25 µM of a synthetic peptide, KKGCVVIVGHIELGK (purchased from LifeTein LLC, US), representing the core of the NS4A cofactor required for the catalytic activity [34, 35]. For the FRET assay, 1 nM of the full-length NS3 protease was used. In the case of NS3-NS4A, the protein (0.5 nM) was incubated in the reaction buffer at 30 °C for 10 min (without the synthetic peptide). The reaction was initiated by the addition of the substrate (10 µL) in a two-fold serial dilution up to a concentration of 2 µM. Reaction wells without the substrate were used as negative controls. For correction of the inner filter effect, a previously described procedure was followed [36]. Kinetic parameters (such as kcat, Km, and kcat/Km) were calculated using the Michaelis–Menten equation. Data were fitted to the equation by non-linear regression using the GraphPad Prism® software (version 7.04, GraphPad Prism®, Inc., USA).

### Validation of FRET assay

For validation of the FRET assay, commercially available inhibitors (asuanaprevir, ciluprevir, danoprevir, and telaprevir (purchased from AdooQ® Bioscience, US) were used to inhibit the activity of the target enzyme (NS3-NS4A protease of HCV genotype-3a), as described previously [37]. Briefly, 1 µM of the depsipeptide substrate was added to the protein, pre-incubated with a given inhibitor, and fluorescence measurements were recorded for 20, 30, or 60 min using 355 nm and 510 nm as excitation and emission wavelengths, respectively. The IC_50_ value, representing the half-maximal inhibitory concentration, of each commercial inhibitor was calculated using the GraphPad Prism® software (version 7.04, GraphPad Prism®, Inc., USA) [38]. The experiment was performed in triplicates, and the average value obtained was considered as the IC_50_ value [39].

For calculation of assay parameters (such as the linearity equation, the R^2^ value, the limit of detection (LOD), and the limit of quantification (LOQ)), a standard curve was drawn between the EDANS concentration (the dye attached with the FRET substrate as a fluorophore) and the fluorescence signal. Moreover, the accuracy and precision of the assay were calculated using telaprevir and danoprevir (commercial inhibitors).

### Preparation of extracts

Extracts from different fruit/seed parts of the following varieties of *Citrus* plants were commercially obtained in a powdered form either from Jiaherb Inc., US or Sanjiang Bio, US: *Citrus aurantinum* [bitter orange], *C. limon* [lemon], *C. paradisi* [grapefruit], *C. reticulata* [mandarin], and *C. sinesis* [orange]. The pomegranate pericarp extract, previously reported to have potent inhibitory activity against HCV NS3 protease [40, 41], was used as a positive control. Four solvents: 70% ethanol in water, ethanol, ethyl acetate, and n-hexane were used to prepare the extracts, as described elsewhere [42]. Briefly, the powdered sample (1 g each) was extracted with 100 mL each of the above-mentioned solvents by shaking (150 rpm) in darkness at ~ 25 °C for 24 h. After filtration of the supernatant, solvents from extracts were evaporated in vacuo using a rotatory evaporator (Buchi R 210) set at 200 rpm and 30 °C. The samples were stored under the cover of Argon or oxygen-free nitrogen amber glass vials in an air-tight environment at -20 °C until further use.

### Evaluation of the inhibitory effect of plant extracts against HCV NS3-NS4A protease

Reaction mixtures (300 µL) were prepared in the assay buffer (50 mM HEPES, pH 7.5, 0.4% Triton X-100, 10 mM DTT, and 40% glycerol). For initial screening, 3.33 mg of the plant extract was dissolved in 1 mL DMSO, and was transferred to the reaction buffer such that the final concentration of DMSO was 3%. The reaction was initiated by adding 0.5 nM NS3-NS4A protease, and the released RET S1 FRET substrate was monitored using a fluorescence microplate reader (TECAN). Reaction mixtures (also containing 3% DMSO) without any plant extract were used for recording blank measurements. The background fluorescence contributed by the plant extract was also recorded. For calculation of the calibration curve, spiking with known amounts (0.25 µM, 0.5 µM, 1 µM, and 2 µM) of the free FRET substrate (RET S1) was made while keeping the reaction mixture at the same volume, containing all the constituents (the plant extract, the assay buffer as well as the enzyme). Slope of the curve was correlated with that obtained from free FRET substrate concentrations without any plant extract as well as the substrate. Initial reaction rates were calculated, and the percentage (%) enzyme inhibition was calculated using the equation: 100 x [a-b/a] where “a” refers to the fluorescence value generated by the reaction mixture only (without the plant extract) and “b” is the solution fluorescence in the presence of the plant extract.

For calculation of the IC_50_ values, extracts were tested at a final three-fold dilution ranging from 1.56 µg/mL to 3.33 mg/mL.

### Mass spectrometric analysis of extracts

Based on the kinetic studies, four *Citrus* extracts (*C. paradisi* (mesocarp), *C. sinesis* (fruit), *C. aurantinum* (fruit), and *C. reticulata* (fruit)) which exhibited a strong inhibitory effect on the activity of NS3-NS4A protease were selected for a detailed investigation of their content using a mass spectrometer (LTQ XL Linear Ion Trap, Thermo Fisher Scientific, Waltham, MA, United States), furnished with an Electrospray Ionization (ESI) probe. Each extract (~ 5 mg) was dissolved in LC–MS grade methanol (5 mL), after passing through a PTFE membrane filter (0.45 µm), were injected to ESI–MS using Direct Syringe Pump while keeping the flow rate at 10 µL min^−1^. The sample scanning was done at negative as well as positive ionization modes by selecting the range of m/z 50–2,000. At the positive ion mode, capillary and source voltages were set at 35 kV and 4.2 kV, respectively, whereas in the negative ionization mode, these values were adjusted at -30 kV and -4.5 kV, respectively. Various parameters, i.e., capillary temperature (280 °C), sheath gas flow rate (N2) (25 L.min^−1^), auxiliary gas flow rate (5 L min^−1^) were adjusted at both ionization modes for the full scan and the tandem mass spectrometric analysis of extract samples. The ion peaks were fragmented through collision induced dissociation (CID) energy value set at 25 (percentage of 5 V) or otherwise stated. The data obtained through full scan MS as well as tandem MS, were processed using the Xcalibur software. Chemical structures (parent and daughter ion peaks) were drawn using the ChemBioDraw Ultra 14.0 software. Compounds were identified by correlating their finger printing fragments with reference standards and published data. The positive control (the pomegranate pericarp extract) was also analyzed as described above.

### Molecular modeling and docking studies of selected compounds

The interaction of NS3-NS4A with compounds identified by ESI–MS/MS was analyzed by molecular docking using the Molecular Operating Environment (MOE) software, as described previously [43, 44]. The ChemBioDraw Ultra 14.0 software was used to prepare chemical structures of compounds identified through the ES-MS/MS analysis. As a three-dimensional structure of HCV NS3-NS4A is currently not available in Protein Data Bank (PDB), a three-dimensional model of the protein was predicted using the SWISS-MODEL online tool [45]. Standard MOE parameters were used for protonation and energy minimization of the generated model. Compounds identified by ESI–MS/MS were docked into the active site of NS3-NS4A using default MOE parameters. Docked poses were analyzed based on S-core values for aromatic stacking and hydrogen bonding interactions [46].

### Evaluation of pure compound (hesperidin)

Based on ESI–MS/MS and molecular docking analyses, a pure natural product (hesperidin, obtained from Jiaherb, USA) in a range of 1230 µg/mL to 1.67 µg/mL was selected and evaluated for inhibition of NS3-NS4A, as described above.

## Results

### Heterologous expression and purification of NS3-NS4A

**Fig. 1:**
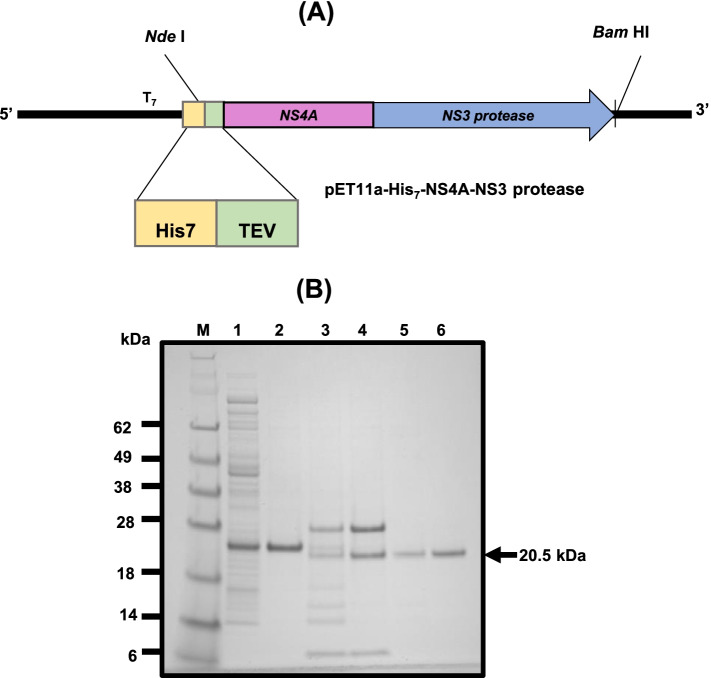
Cloning strategy, expression, and purification of NS3-NS4A of HCV genotype 3a. **A** The codon-optimized nucleotide sequence encoding NS3 protease domain fused to the NS4A cofactor was cloned in the pET11a plasmid under the control of the *T7* promoter using NdeI and BamHI restriction sites. The construct was used for heterologous expression of NS3-NS4A fused to a polyhistidine (His_7_) tag in *E. coli*. **B** The soluble form of NS3-NS4A expressed in *E. coli* BL21 (DE3) cells was analyzed through SDS-PAGE. Lane 1 represents the soluble fraction of the cell lysate (from the cells induced with IPTG) analyzed on a 4–12% Bis–Tris NuPAGE gel. The protein in the native form was successfully purified through affinity chromatography and gel filtration. Lane 2 shows the sample collected from the HisTrap column during the elution step, lanes 3 and 4 depict the digestion mixture (containing His-tagged AcTEV protease and NS3-NS4A), lane 5 represents the sample collected during reverse affinity chromatography, and lane 6 shows the sample collected after gel filtration. Lane M represents the mobility of proteins with known molecular weights (SeeBlue Pre-stained Protein Marker)

The expression analysis through SDS-PAGE revealed that His_7_-NS3-NS4A was expressed in a highly soluble form in *E. coli* BL21 (DE3) cells using the pET11a-His_7_-NS3-NS4A plasmid (Fig. 1). The expressed protein was purified through immobilized metal affinity chromatography using His-Trap columns, and impurities, if any, were removed through gel filtration. The oligohistidine tag at the N-terminus of the protein was successfully removed using TEV protease, and, after reverse affinity chromatography and gel filtration, the protein was found to be more than 95% pure as analyzed through SDS-PAGE (Fig. 1B). The obtained purification yield of NS3-NS4A was 6 mg per liter of the bacterial culture.

### Activity analysis of NS3-NS4A through FRET assay

**Fig. 2:**
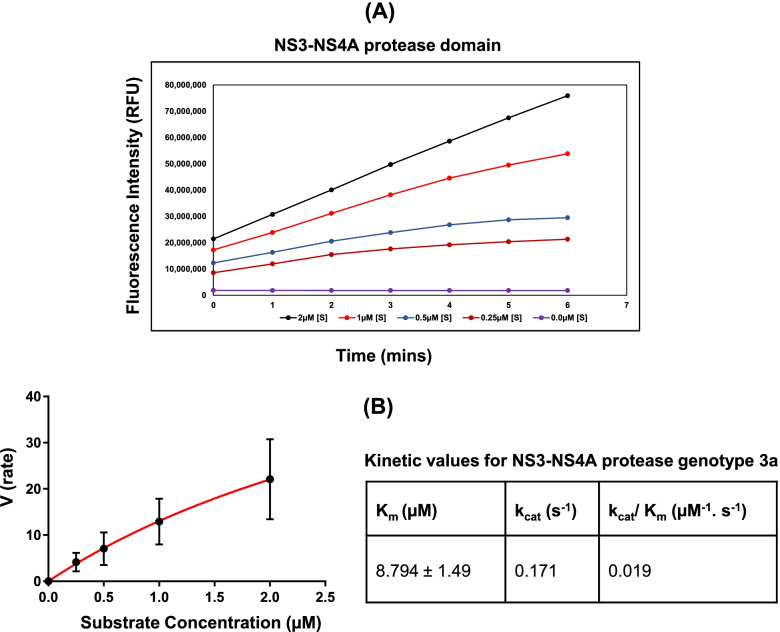
Activity analysis of purified NS3-NS4A. **A** The activity of NS3-NS4A increased upon the addition of the depsipeptide substrate, indicated by a corresponding increase in the fluorescence intensity. **B** Kinetic parameters (Km, kcat, and kcat/Km) calculated for NS3-NS4A using the Michaelis Menten equation are presented

A concomitant increase in the activity of the fusion complex (NS3-NS4A protease) was observed upon increasing the concentration of the depsipeptide substrate in the FRET assay (Fig. 2). However, a corresponding increase in the activity was not observed when the full-length His_6_-NS3 was used in the assay (see Supplementary Materials, Figure S1 & S2), suggesting that NS3-NS4A represents a catalytically active complex that could be employed for enzyme inhibition studies. Furthermore, kinetic parameters calculated for NS3-NS4A (Fig. 2B) indicated a very high substrate binding affinity (Km 8.79 µM) as well as a high catalytic efficiency (kcat/Km 0.019 µM^−1^.s^−1^), suggesting that NS3-NS4A is suitable for conducting inhibitor screening assays.

The FRET assay was validated using commercially available inhibitors (asuanaprevir, ciluprevir, danoprevir, and telaprevir), and IC_50_ values in the nanomolar range were obtained (Table 1), confirming that reaction conditions for the FRET assay are properly optimized and are suitable for conducting inhibition studies using natural extracts/compounds from *Citrus* plants.

**Table 1:** Half-maximal inhibitory concentration (IC_50_ values) calculated for commercial inhibitors of HCV NS3 protease using the FRET assay

Sr. No	Inhibitor	IC_50_ value (µg/mL)
1	Telaprevir	0.0475 ± 0.0075
2	Danoprevir	0.0184 ± 0.0036
3	Asunaprevir	0.0511 ± 0.0093
4	Ciluprevir	0.0412 ± 0.0101

Various parameters (such as accuracy, precision, the limit of detection (LOD), and the limit of quantitation (LOQ)) calculated for the developed assay are given in Tables S1-S4 & Figure S3.

### FRET-based screening of *Citrus* extracts

**Fig. 3:**
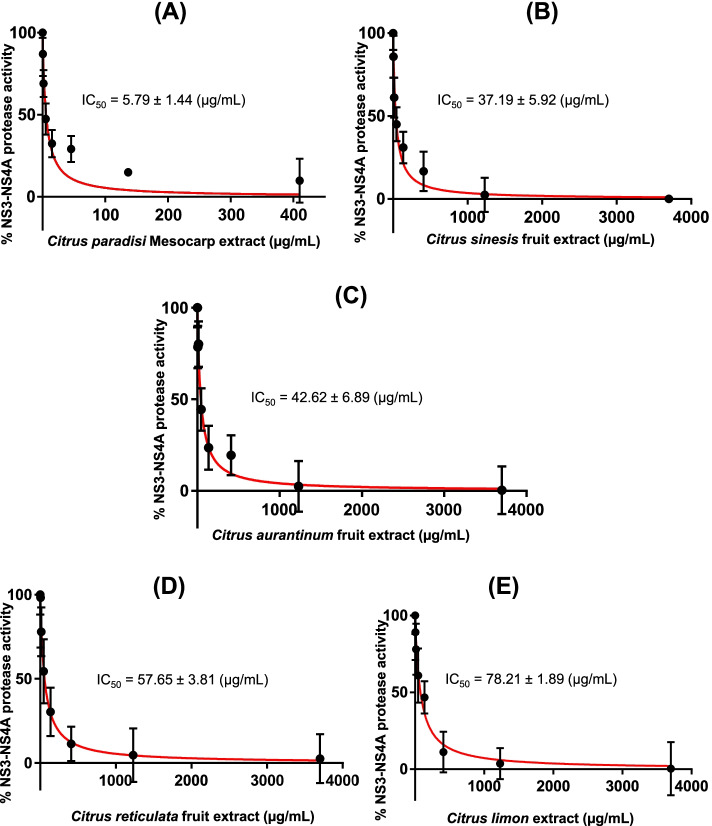
Inhibitory effect of *Citrus* extracts on the activity of NS3-NS4A. *Citrus* extracts significantly inhibited the activity of NS3-NS4A as measured through the validated FRET assay. The maximum inhibitory effect was measured for the *C. paradisi* mesocarp extract (IC_50_ value of 5.79 ± 1.44 µg/mL; **A**, followed by the *C. sinesis* fruit extract **B**, the *C. aurantinum* fruit extract **C**, the *C. reticulata* fruit extract **D**, and the *C. limon* fruit extract **E**

FRET-based screening of 14 extracts from *Citrus* plants revealed that the maximum inhibition (IC_50_ value of 5.79 ± 1.44 µg/mL) of NS3-NS4A protease was exhibited by the *C. paradisi* mesocarp extract, followed by fruit extracts of *C. sinesis* (IC_50_ value of 37.19 ± 5.92 µg/mL), *C. aurantinum* (IC_50_ value of 42.62 ± 6.89 µg/mL), *C. reticulata* (IC_50_ value of 57.65 ± 3.81 µg/mL), and *C. limon* (IC_50_ value of 66.83 ± 4.92 µg/mL), while the IC_50_ values obtained for the remaining extracts ranged from 71.94 ± 2.39 µg/mL to 196.40 ± 2.92 µg/mL (Table 2). All the afore-mentioned extracts displayed IC_50_ values in the micro-molar range, suggesting that these natural extracts contain potent inhibitors of HCV NS3-NS4A protease (Fig. 3, Table 2). The pomegranate pericarp extract (used as a positive control in the current study) significantly inhibited the activity of NS3-NS4A, exhibiting an IC_50_ value of 5.52 ± 0.74 µg/mL (Figure S4**)**.

**Table 2:** Inhibitory effect of *Citrus* extracts on the activity of NS3-NS4A. The extracts were screened using the validated FRET assay, percentage inhibition and IC_50_ values were calculated for each extract. The pomegranate pericarp extract was used as a positive control

Sr. #	Scientific name	Common name	Plant parts used	% Inhibition ($\frac{{\varvec{a}}-{\varvec{b}}}{{\varvec{a}}}$*100)	IC_50_ value (µg/mL)
1	*Citrus paradisi*	Grapefruit	Mesocarp	91%	5.79 ± 1.44
2	*Citrus sinesis*	Orange	Fruit	86%	37.19 ± 5.92
3	*Citrus aurantinum*	Bitter Orange	Fruit	85%	42.62 ± 6.89
4	*Citrus reticulata*	Mandarin	Fruit	82%	57.65 ± 3.81
5	*Citrus limon*	Lemon	Fruit	80%	66.83 ± 4.92
6	*Citrus aurantinum*	Bitter Orange	Pericarp	79%	71.94 ± 2.39
7	*Citrus aurantinum*	Bitter Orange	Mesocarp	78%	77.06 ± 4.26
8	*Citrus reticulate*	Mandarin	Seeds	77%	80.02 ± 3.67
9	*Citrus sinesis*	Orange	Seeds	75%	86.93 ± 5.16
10	*Citrus reticulate*	Mandarin	Mesocarp	74%	90.59 ± 1.89
11	*Citrus paradisi*	Grapefruit	Pericarp	71%	107.90 ± 5.64
12	*Citrus paradisi*	Grapefruit	Fruit	69%	120.30 ± 2.08
13	*Citrus paradisi*	Grapefruit	Seeds	68%	132.23 ± 2.12
14	*Citrus aurantinum*	Bitter Orange	Seeds	63%	196.40 ± 2.92
15	*Punica granatum*	Pomegranate	Pericarp	98%	5.52 ± 0.74

### Mass spectrometric analysis of *C. paradisi* (mesocarp), *C. sinesis *(fruit), *C. aurantinum* (fruit) and *C. reticulata* (fruit) extracts

**Fig. 4:**
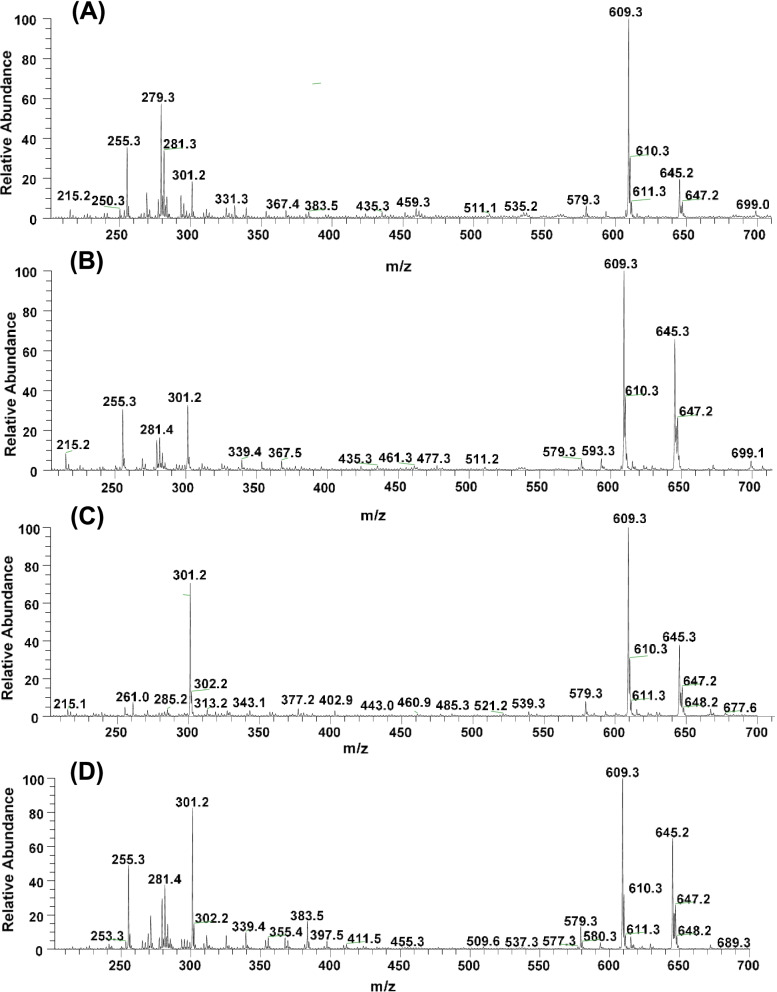
Full scan LC–MS analysis of top 4 potentially active *Citrus* extracts. **A** *C. paradisi* mesocarp extract, **B** *C. sinesis* fruit extract, **C** *C. aurantinum* fruit extract, and **D** *C. reticulata* fruit extract

Out of 14 *Citrus* extracts, the top four active extracts against NS3-NS4A protease domain, i.e., *C. paradisi* mesocarp, fruit extracts of *C. sinesis*, *C. aurantinum* and *C. reticulata* were analyzed by ESI-MS^n^ for the identification of active natural compound(s). Various solvents (80% ethanol, 100% ethanol, ethyl acetate and *n-*hexane) were used to prepare extracts and among these, *n*-hexane and ethyl acetate gave better results in curtailing the noise and higher intensities of polyphenol peaks. The results related to identification of compounds in these extracts by ESI-MS^n^ have been summarized in Table 3. The full scan mass spectrum at the negative ion mode of the *C. paradisi* mesocarp extract gave several ion peaks in the range of *m/z* 100—700 (Fig. 4A, Table 3). To precisely identify natural products in extracts, each peak in negative [M-H]^1−^ as well as positive [M + H]^1+^ ion modes was subjected to collision induced dissociation (CID) lead fragmentation. The ESI-MS^n^ analysis [M-H]^1−^ discovered the presence of bergapten (*m/z* 215), aliphatic/aromatic organic acids (*m/z* 255, 279, 281, 283, 367, 383 and 459); aglycone flavonoids, i.e., alpinetin (*m/z* 269), hesperitin (*m/z* 331) and methoxyflavone (*m/z* 435); coumarin, i.e., (R)-marmin (*m/z* 331) &; several glycone flavonoids such as naringenin arabinofuranose (*m/z* 535, 2% abundance), naringin (*m/z* 579, 6% abundance), hesperidin (*m/z* 609, 100% abundance), neohesperidin (*m/z* 610, 29% abundance), di-hydrated adduct of hesperidin [M-H + 2H_2_O]^−1^ m*/z* 645, ~ 19% abundance) and traces of di-hydrated adduct of cyanidin-3-O-sophoroside chloride [M-2H + 2H_2_O]^−1^. The extracts were also analyzed at the positive ionization mode [M + H]^1+^, which revealed the identification of predominantly methoxylated flavonoids, i.e., limonene, synephrine, and tangeretin. Structures of the identified natural products were confirmed by the ESI-MS^n^ analysis.

**Table 3:** The identified compounds and their relative abundance in top 4 active *Citrus* extracts

*m/z* (-ve ion mode)	Identified compounds name	Relative abundance (%) of metabolites in top 4 active *Citrus* extracts			
		***C. paradisi***^**a**^	***C. sinesis***^**b**^	***C. aurantinum***^**c**^	***C. reticulata***^**d**^
215	Bergaptene	4	0	0	0
215	Gallic acid	0	8	4	0
255	Palmitic acid	34	32	0	51.5
269	Alpinetin	12	0	0	0
279	Linoleic acid	56	0	0	0
281	Octadec-12-enoic acid	33	16	0	37.5
283	Stearic acid	12	0	0	0
301	Hesperitin	18	38	12	83
301	Quercetin	0	0	88	0
331	(R)-Marmin	7	0	0	0
339	Esculin	0	7	0	10
367	Lignoceric acid	4	7	0	0
377	Galactinol dihydrate	0	0	5	0
383	Cerebronic acid	3	0	0	16
435	Methoxyflavone	3	2	0	0
459	Feruric acid di-saccharide	6	0	0	0
461	Diosmetin-7-O-glucoside	0	2	0	0
477	Isorhamnetin glucoside	0	0	5	0
535	Naringenin arabinofuranose	2	0	0	0
539	Sugar oligomers (trisaccharides and their water adducts)	0	0	3	0
579	Naringin	6	6	8	11
593	Poncirin	0	6	0	0
609	Hesperidin	100	100	100	100
610	Neohesperidin	29	36	32	32
645^e^	i-Hesperidin + 2H_2_O ii-Cyanidin-3-O-sophoroside chloride	20	64	41.5	66
647	Rutin adduct	8	52	17	37

^a^Grapefruit mesocarp extract,^b^Orange whole fruit extract,^c^Bitter orange whole fruit extract, and ^d^Mandarin whole fruit extract.^e^Cyanidin-3-O-sophoroside chloride is only detected in traces in *C. paradisi* n-hexane extract, *m/z* 645 predominantly belongs to the dihydrated adduct of hesperidin in all extracts

Similarly, compounds were identified in fruit extracts of *C. sinesis*, *C. aurantinum* and *C. reticulata* by the ESI-MS^n^ analysis, which gave more or less similar profile patterns of secondary metabolites as spotted in the *C. paradisi* extract (Fig. 4 & Table 3), except that alpinetin (*m/z* 269), (R)-marmin (*m/z* 331), feruric acid di-saccharide (*m/z* 459) and naringin arabinofuranose (*m/z* 535) were only identified in *C. paradisi* extract. Whereas, hesperitin (*m/z* 331) was present in significantly higher abundance in *C. reticulata*, quercetin (*m/z* 301) and isorhamnetin glucoside (*m/z* 477) were only spotted in *C. aurantinum*, esculin (*m/z* 339) and diosmetin-7-O-glucoside (*m/z* 461) were located in *C. sinesis*.

**Fig. 5:**
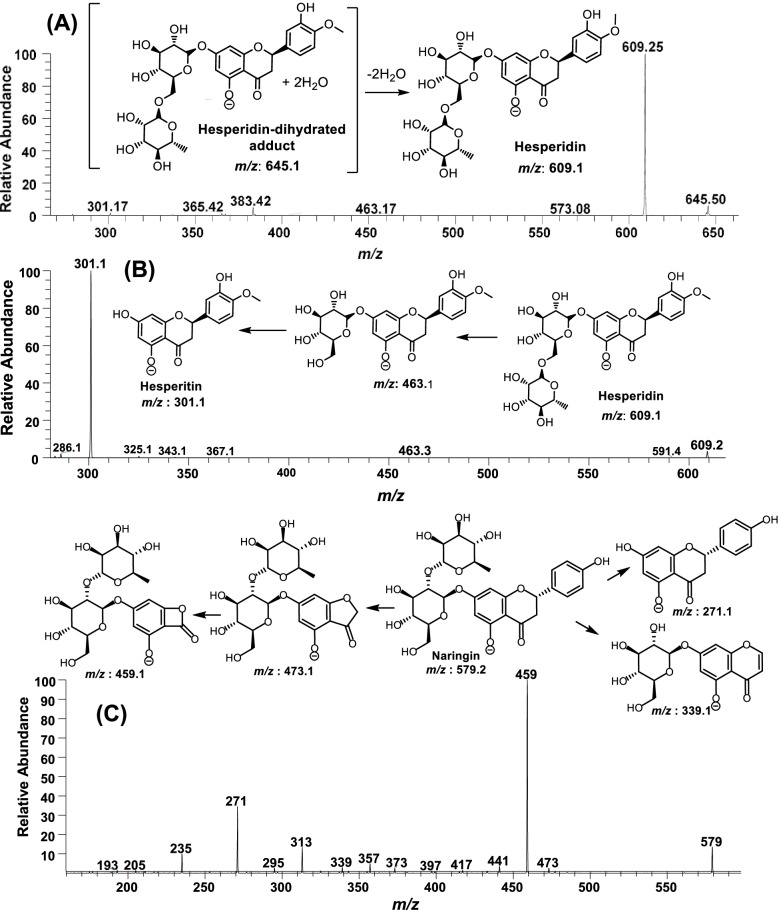
MS^2^ analysis **A** hesperidin-dihydrate (*m/z* 645) @CID 3.5, **B** hesperidin *(m/z* 609) @CID 3.5, and **C** naringin @CID 3.5

All four extracts exhibited a high abundance of hesperidin (*m/z* 609) and hesperidin di-hydrated adduct (*m/z* 645) (Table 3, Fig. 4). Structures of both the compounds were determined through tandem mass spectrometry. The ion peak *m/z* 645 was fragmented @CID 3.5 at the negative ion mode, which after losing 2H_2_O (two molecules of water involved in adduct formation) gave peak *m/z* 609 as a base peak (Fig. 5A), further MS^3^ fragmentation of *m/z* 609 yielded a minor daughter ion (*m/z* 463) after losing one hexose and a major daughter ion (*m/z* 301) by dissociating both hexoses. Notably, the native ion peak *m/z* 609 produced during the full scan of all four extracts as well the daughter ion (*m/z* 609) of *m/z* 645 gave a similar fragmentation pattern as described in Fig. 5B. Naringin (*m/z* 579) was also universally present in all *Citrus* extracts and its fragmentation produced the signature daughter ions, i.e., *m/z* 473, 459, 339 and 271 ion peaks (Fig. 5C).

**Fig. 6:**
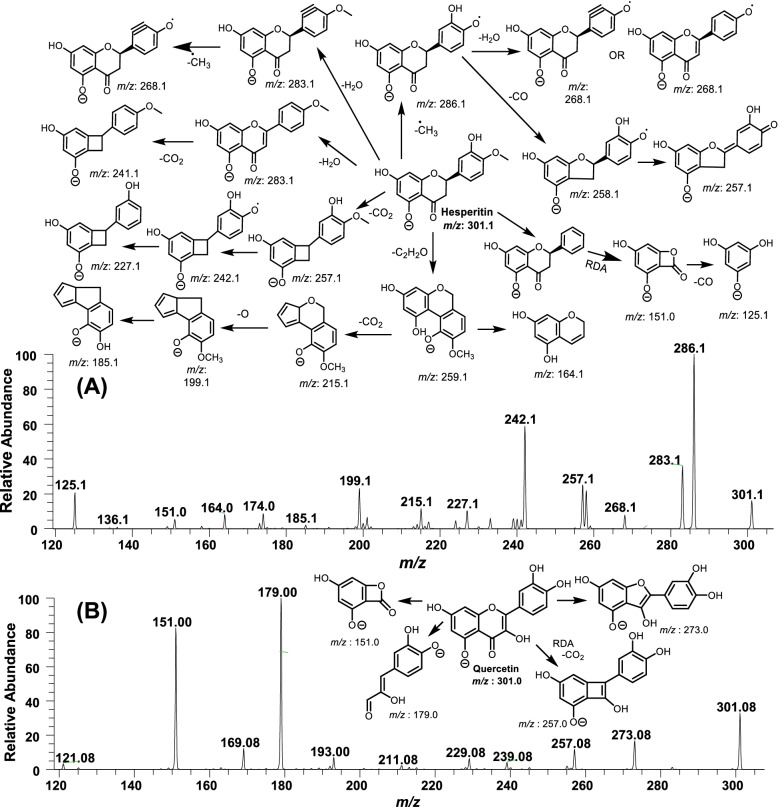
MS^n^ analysis of flavonoids, **A** MS^2^ fragmentation of hesperitin and MS^3^ fragmentation of hesperidin, and **B** MS^2^ fragmentation of quercetin

Among the aglycone flavonoids, hesperitin and quercetin exhibit the same ion peak at *m/z* 301. The tandem mass spectrometric analysis enabled us to differentiate and precisely elucidate the structures of hesperitin and quercetin metabolites. Ion peaks produced by each of the four *Citrus* extracts at *m/z* 301 were subjected to fragmentation, which yielded two types daughter ion peaks patterns as described in Fig. 6**.** The *C. paradisi* mesocarp extract and fruit extracts of *C. sinesis* and *C. reticulata* yielded the daughter ions matching the hesperitin fragmentation pattern (Fig. 6A). Whereas, *C. aurantinum* ion peak (*m/z* 301) produced the daughter ion, which correlated with quercetin predominantly (~ 90%) (Fig. 6B), and only traces of hesperitin daughter ions were spotted in this extract.

Considering the detailed MS^2^ fragmentation analysis of hesperitin (*m/z* 301) isolated from full scans as well as the daughter ion of *m/z* 609 (MS^3^) (@CID3.5), yielding several daughter/grand-daughter peaks with *m/z* 286 being a base peak produced by the loss of methyl radical (-CH_3_•), which subsequently generated ion peaks by losing a -H_2_O (*m/z* 268), -CO (*m/z* 258) and rearrangement of a double bond (*m/z* 257) (Fig. 6A). The subsequent fragmentation of *m/z* 258 yielded *m/z* 151 and *m/z* 125 by losing the B and the C rings, respectively. The ion peak *m/z* 301 fragmentation further produced *m/z* 283 after losing H_2_O, subsequently yielding the alkyne adduct and C ring extended conjugation, which after losing CO_2_ formed an ion peak at *m/z* 241. The ion peak *m/z* 268 was produced from alkyne adduct (*m/z* 283) after losing a -CH_3_•. Whereas, hesperitin (*m/z* 301) also produced *m/z* 227, *m/z* 242 and *m/z* 257 after losing O•, CH_3_• and CO_2_, respectively. The *m/z* 151 may likely to be produced directly after fragmentation of *m/z* 301 and/or its subsequent adducts bearing C ring possibly through Retro Diels–Alder reactions. Markedly, the ion peaks *m/z* 151 and *m/z* 125 are considered as the signature ion fragments produced by most of the flavonoids during the ESI-MS^n^ analysis [47]. Moreover, *m/z* 301 fragmentation yielded fused ring products (*m/z* 259, 215, 199 and *m/z* 185). All of the data analysis of daughter ion peaks confirmed the m/z 609 as a hesperidin structure [47, 48]. To further confirm the structure of ion peak at *m/z* 609, the fragmentation of authentic hesperidin standard data was found to be the fully correlated with fragmentation pattern of peak identified in the extracts of the *C. paradisi* mesocarp extract and fruit extracts of *C. sinesis* and *C. reticulata*.

Fragmentation of the ion peak *m/z* 301 spotted in *C. aurantinum* yielded the signature daughter ions of quercetin, i.e., *m/z* 273, 257, 179 and 151 ion peaks (Fig. 6B), which fully correlated with peaks produced during fragmentation of the quercetin reference standard.

The detailed mass spectrometric analysis of the pomegranate pericarp extract (used as a positive control for inhibition of NS3-NS4A in the current study) can be found in Figure S5**.**

### Molecular modeling and docking studies of selected compounds

A good quality model of NS3-NS4A (with excellent stereochemical properties such as 98% residues in the most favored region of the Ramachandran plot, a QMean score of -1.77, and an overall quality factor of more than 93% as measured through the ERRAT software) allowed us to use the generated homology model for docking studies with compounds identified through the ESI–MS/MS analysis.

**Fig. 7:**
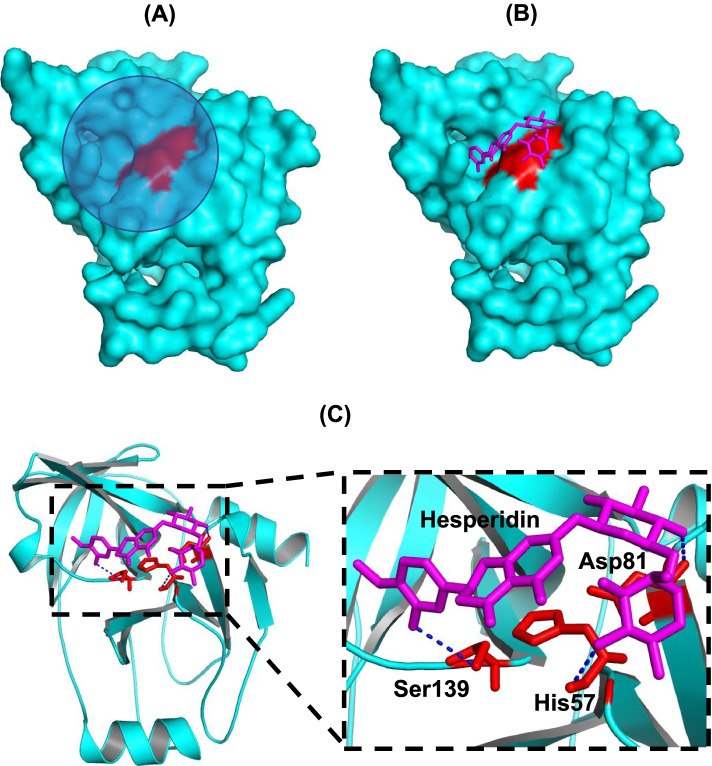
Docking of hesperidin in the active site of NS3 protease. **A** The region in the vicinity of active site residues (His57, Asp81, and Ser139; colored red) selected for docking of hesperidin is depicted with a blue circle. The receptor protein (NS3 protease of HCV genotype 3a) is colored in cyan. **B** The complex consisting of the receptor protein (colored cyan) and the docked molecule (hesperidin; shown as magenta sticks) in the vicinity of active site residues (colored red) is shown. **C** The docking complex showing the interaction of hesperidin (shown as magenta sticks) with active site residues (shown as red sticks) is presented. It is suggested that hesperidin inhibits NS3 protease by forming hydrogen bonds (shown as blue dotted lines) with active site residues (His57, Asp81, and Ser139)

Results obtained from the docking experiment suggest that the highest affinity for NS3-NS4A protease is exhibited by hesperidin (S-score value of -10.98) among the identified compounds. Hesperidin also interacts with the catalytic triad of the enzyme (Fig. 7), suggesting that hesperidin is a potent inhibitor of NS3-NS4A protease.

### Evaluation of pure flavonoids

**Fig. 8:**
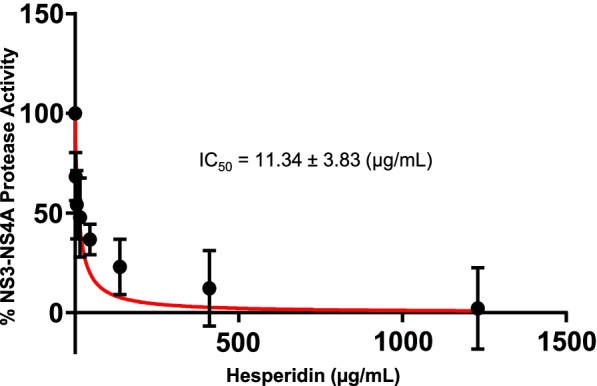
Inhibitory effect of hesperidin on the activity of NS3-NS4A. Hesperidin potently inhibited the activity of NS3-NS4A as measured through the FRET assay, yielding an IC_50_ value of 11.34 ± 3.83 µg/mL

The ability of hesperidin to inhibit the activity of NS3-NS4A was confirmed using the FRET assay. It was observed that commercially obtained hesperidin significantly inhibited the activity of NS3-NS4A (with an IC_50_ value of 11.34 ± 3.83 µg/mL (Fig. 8), confirming that hesperidin is a potent inhibitor of the enzyme. Ellagic acid (Jiaherb, USA), identified from the pomegranate pericarp extract (positive control) (Figure S6- S8)**,** exhibited an IC_50_ value of 29.62 ± 1.47 µg/mL against HCV NS3-NS4A protease (Figure S9).

## Discussion

Despite the availability of effective direct-acting anti-HCV drugs, HCV infections are continuously increasing across the globe [3]. This is in part due to the high cost of the approved drugs, making it difficult for HCV-infected individuals, particularly from resource-limited and developing countries, to access the treatment option. Moreover, HCV, similar to other RNA viruses, is prone to mutations due to the lack of a proof-reading activity of its RNA polymerase, ultimately leading to the emergence of resistant HCV variants to currently available drugs [4]. Hence, there is a need to develop new anti-HCV drugs in order to combat HCV infections.

In the current study, we selected NS3 protease of HCV genotype 3a as a drug target due to its pivotal role in processing of viral proteins [11]. In order to identify potent inhibitors of NS3 protease, we employed the purified NS3 protease from HCV genotype 3a in fusion with its cognate NS4A cofactor in a FRET assay for screening of natural extracts from *Citrus* plants. Initially, a FRET assay was established using the purified NS3 protease of HCV genotype 3a. As expected, our efforts to establish a FRET assay using His_6_-NS3 protease alone (without a fused NS4A cofactor) did not prove fruitful (Figure S2). However, the fusion complex (NS3-NS4A) displayed a significantly high catalytic activity in the presence of a depsipeptide substrate in the FRET assay, suggesting that HCV genotype 3a NS3 is catalytically active only in fusion with the NS4A cofactor (Fig. 2). The FRET assay was validated using commercially available inhibitors (asuanaprevir, ciluprevir, danoprevir, and telaprevir) which significantly decreased the activity of the enzyme (Table 1). Furthermore, the IC_50_ values of commercial inhibitors (such as telaprevir (0.0475 ± 0.0075 µg/mL) and danoprevir (0.0184 ± 0.0036 µg/mL)) obtained in the current study are very close to the IC_50_ values (0.0475 µg/mL and 0.0146 µg/mL, respectively) reported elsewhere [49, 50] (Table S3**).** In agreement with previous studies [40, 41], the positive control (the pomegranate pericarp extract) significantly inhibited the activity of HCV NS3-NS4A, displaying an IC_50_ value of 5.52 ± 0.74 µg/mL (Figure S4). The validated FRET assay was used for screening of natural extracts from *Citrus* plants, which are rich in antiviral compounds such as flavonoids [29–31]. Inhibitory activities of flavonoids are well-documented against multiple viruses such as herpes simplex virus [51], respiratory syncytial virus [52, 53], poliovirus [54, 55], sindbis virus [56], and dengue virus [57]. Our selection of *Citrus* plant extracts was based on previous studies, which demonstrated that *Citrus* plants are a rich source of flavonoids (such as apigenin, quercetin, and naringenin) exhibiting anti-HCV activities [29–31]. Our screening campaign revealed that the *C. paradisi* mesocarp extract displayed the lowest IC_50_ value (5.79 ± 1.44 µg. mL^−1^), against NS3-NS4A protease of HCV genotype 3a out of 14 *Citrus* fruits extracts tested in the current study **(**Fig. 3A, Table 2).

Subsequent analysis by LC–MS/MS revealed hesperidin as the most abundant constituent of the *Citrus* extract **(**Fig. 4, Table 3**)**. Structures of hesperidin, hesperitin, and quercetin were confirmed through the ESI-MS^n^ technique **(**Fig. 5 & 6**)**. Notably, the orange extract, the bitter orange extract, and the mandarin extract exhibited the 2^nd^, 3^rd^ and 4^th^ best inhibitory activity against NS3-NS4A protease **(**Fig. 3B, 3C & 3D**)**, and the afore-mentioned extracts also revealed the presence of hesperidin, as demonstrated by the ESI–MS analysis **(**Fig. 4&Table 3**)**. In order to confirm that hesperidin is a potent inhibitor of NS3-NS4A, commercially available hesperidin (90% pure; obtained from Jiaherb, USA) was evaluated in the FRET assay. In agreement with results obtained from screening of *Citrus* extracts, hesperidin inhibited the activity of NS3-NS4A with high potency.

Notably, the broad-spectrum antiviral activity of hesperidin has previously been reported against a range of viruses such as poliovirus, parainfluenza virus, herpes simplex virus, vesicular stomatitis virus, vaccinia virus, rotavirus, human immunodeficiency virus, canine distemper virus, hepatitis B virus, and sindbis virus [56, 58–63]. Recently, it has been shown that hesperidin actively inhibits SARS-CoV-2 replication in the cell culture model [64]. Hesperidin is one of the safest flavonoids for human use that can help i) normalize glucose metabolism and mitigate the diabetes mellitus, ii) alleviate hyperlipidemia, iii) diminish retinal and plasma abnormalities, iv) improve liver inflammation and hepatocellular steatosis, and v) prevent cardiovascular diseases [65–71]. The pharmacological effects of hesperidin are also due to its ability to scavenge free radicals, and it has been demonstrated that hesperidin also plays an important role in cutaneous wound healing, skin whitening by inhibiting melanogenesis, and protection against Alzheimer’s disease and various types of cancers [63, 72–76].

Natural compounds with demonstrated inhibitory potential against HCV NS3 protease include excoecariphenol D corilagin, 3-hydroxy caruilignan C, quercetin, epigallocatechin-3 gallate, and honokiol [30, 77–82]. However, hesperidin displays the lowest IC_50_ value 11.34 ± 3.83 µg/mL (Fig. 8) compared to the above-mentioned compounds. The IC_50_ value of hesperidin against HCV NS3-NS4A is better than that of ellagic acid (29.62 ± 1.47 µg/mL; Fig. 8 & S9), the bioactive compound identified in the pomegranate extract [40, 41]. In order to gain insights into the molecular interaction of hesperidin with HCV NS3 protease, molecular docking studies were performed, which suggested that hesperidin inhibits NS3 protease by blocking active site residues (Fig. 7).

Among the tested *Citrus* extracts, the highest IC_50_ value against NS3-NS4A is exhibited by the bitter orange seeds extract(Table 2). The LCMS analysis of the bitter orange seeds extract reveals that the weak inhibitory effect of the extract against HCV NS3-NS4A protease could be attributed to a very low relative abundance of hesperidin in the extract (Figure S10). Moreover, the docking analysis of the other identified compounds (palmitic acid, linoleic acid, and cerebronic acid) from the bitter orange seeds extract suggests that these compounds do not interact with the active site residues (the catalytic triad: His57, Ser139, and Asp81), except linoleic acid which interacts only with His57 from the catalytic triad (Figure S11). In contrast, hesperidin and telaprevir inhibit the activity of HCV NS3-NS4A protease by blocking the active site residues (Fig. 7 & Figure S11**). **As hesperidin is naturally present in abundant quantities in grapefruit and other plants, especially in the *Citrus* family, hence, hesperidin could be pursued as a cost-effective drug candidate against HCV genotype 3a.

## Conclusions

In the current study, we demonstrated that NS3 protease from HCV genotype 3a is functionally active in the NS4A-fused form, which can be employed for the development of a FRET assay for the discovery of anti-HCV inhibitors. Using a validated FRET assay, plant extracts from the *Citrus* family were evaluated for their inhibitory effect on the activity of NS3-NS4A. Results of the FRET assay together with ESI–MS/MS and molecular docking analyses revealed that a flavonoid, hesperidin which is abundantly present in the grapefruit mesocarp, orange, bitter orange, and mandarin whole fruit extracts, could act as an effective inhibitor of HCV NS3-NS4A protease, suggesting that *Citrus* fruit extracts are a rich source of cost-effective natural products with antiviral bioactivities.

## Supplementary Information

**Additional file1:Figure S1.** Cloning strategy, expression and purification of the full-length NS3 of HCV genotype 3a. (A) The gene encoding the full-length NS3 was cloned in the pET11a plasmid under the control of the T7 promoter using BamHI and HindIII restriction sites. The construct was used for heterologous expression of the full-length NS3 fused to a polyhistidine (His_6_) tag in *Escherichia coli*. (B) Expression analysis of the full-length NS3 in *E. coli* BL21 (DE3) cells (lanes 1 and 2). The cell lysate was analyzed on a 4-12% Bis-Tris NuPAGE gel. Lanes 1 and 2 represent the soluble fraction of the cell lysate from the uninduced cells and IPTG-induced cells, respectively. Lanes 3 to 9 represent samples not related to the current study. The black arrow indicates the band corresponding to the estimated molecular weight (~ 68 kDa) of the full-length NS3. Lane M represents the mobility of proteins with known molecular weights (SeeBlue Pre-stained Protein Marker). (C) Expression analysis of the full-length NS3 in BL21-CodonPlus(DE3)-RIL cells (lanes 1 and 2). The cell lysate was analyzed ona 4-12% Bis-Tris NuPAGE gel. Lanes 1 and 2 represent the soluble fraction of the cell lysate from the uninduced cells and IPTG-induced cells, respectively. Lane 3 represents the sample collected during stringent washing of the HisTrap column. Lanes 4, 5, and 6 represent the sample collected during the elution step from the HisTrap column, pooled samples after gel filtration, and the last fraction collected during gel filtration, respectively. Samples were analyzed on a 4-12% Bis-Tris NuPAGE gel. **Figure S2. **Activity analysis of purified full-length NS3. Upon increasing the substrate concentration, no detectable increase in the fluorescence intensity was observed in the case of the full-length His_6_-NS3, suggesting that the full-length His_6_-NS3is non-functional in the absence of a fused NS4A cofactor. **Figure S3. **A standard calibration curve plotted between various concentrations of EDANS and the generated fluorescence signal. A linearline having an R^2 ^value of 0.996 was obtained. **Figure S4. **Inhibitory effect of the pomegranate pericarp extract (used as a positive control) on the activity of NS3-NS4A. The pomegranate extract significantly inhibited the activity of NS3-NS4A (IC_50_ value of 5.52 ± 0.74 µg/mL) as measured through the validated FRET assay. **Figure S5. **ESI-MS/MS analysis of *P. granatum* pericarp methanolic extract in negative ion mode. **Figure S6.** Proposed fragmentation of ellagic acid hexoside based on quasi-ESI-MS^n ^spectra in negative ion mode. **Figure S7. **Proposed fragmentation of ellagic acid based on quasi-ESI-MS^n^ spectra in negative ion mode. **Figure S8.** Proposed fragmentation of ellagic acid pentoside based on quasi-ESI-MS^n ^spectra in negative ion mode. **Figure S9. **Inhibitory effect of ellagic acid on the activity of NS3-NS4A. Ellagic acid significantly inhibited the activity of NS3-NS4A as measured through the FRET assay, yielding an IC_50_value of 29.62 ± 1.47 µg/mL. **Figure S10. **ESI-MS/MS analysis of the bitter orange seeds extract in the negative ion mode. **Figure S11.** Docking of strong (telaprevir) and weak (palmitic acid, linoleic acid, and cerebronic acid) inhibitors in the active site of HCV genotype 3a NS3 protease. The region around the active site selected for docking of compounds is depicted as a blue circle. Active site residues (His57, Asp81, and Ser139) are shown in red, while docked compounds are shown as orange sticks (telaprevir), magenta sticks (palmitic acid), green sticks (linoleic acid), and blue sticks (cerebronic acid). Hydrogen bonds are depicted as blue dotted lines. The interaction of telaprevir, palmitic acid, linoleic acid, and cerebronic acid with active site residues is presented in **(A)**, **(B)**, **(C)**, and **(D)**, respectively. **Table S1. **Inhibition trials of telaprevir against NS3-NS4A protease. **Table S2.** Calculation of Linearity, LOD and LOQ using standard curve. **Table S3.** Comparison of IC_50 _values of commercial inhibitors used in study with IC_50s _reported in the literature. **Table S4.** Inhibition trials of danoprevir against NS3-NS4A protease

## Data Availability

The data and raw materials presented in the current study are available from the corresponding author on reasonable request. Commercial inhibitors **(**asuanaprevir, ciluprevir, danoprevir, and telaprevir) were acquired from AdooQ® Bioscience, US. Plant extracts were acquired from Jiaherb Inc., US and Sanjiang Bio, US.

## Abbreviations

HCV: Hepatitis C virus
IC_50_: Half-maximal inhibitory concentration
ESI: Electrospray ionization
FRET: Fluorescence Resonance Energy Transfer
RNA: Ribonucleic acid
WHO: World Health Organization
HEPES: N-2-Hydroxyethylpiperazine-N'-2-Ethanesulfonic Acid
NaCl: Sodium chloride
LC–MS/MS: Liquid chromatography–mass spectrometry
PDB: Protein Data Bank
