# Supplementary material for: Hesperidin identified from Citrus extracts potently inhibits HCV genotype 3a NS3 protease
Source: BMC Complement Med Ther. 2022 Apr 2;22:98. doi: 10.1186/s12906-022-03578-1 (PMC8976278; doi:10.1186/s12906-022-03578-1)
Supplement: Supplementary file 1 — Additional file1:Figure S1. Cloning strategy, expression and purification of the full-length NS3 of HCV genotype 3a. (A) The gene encoding the full-length NS3 was cloned in the pET11a plasmid under the control of the T7 promoter using BamHI and HindIII restriction sites. The construct was used for heterologous expression of the full-length NS3 fused to a polyhistidine (His6) tag in Escherichia coli. (B) Expression analysis of the full-length NS3 in E. coli BL21 (DE3) cells (lanes 1 and 2). The cell lysate was analyzed on a 4-12% Bis-Tris NuPAGE gel. Lanes 1 and 2 represent the soluble fraction of the cell lysate from the uninduced cells and IPTG-induced cells, respectively. Lanes 3 to 9 represent samples not related to the current study. The black arrow indicates the band corresponding to the estimated molecular weight (~ 68 kDa) of the full-length NS3. Lane M represents the mobility of proteins with known molecular weights (SeeBlue Pre-stained Protein Marker). (C) Expression analysis of the full-length NS3 in BL21-CodonPlus(DE3)-RIL cells (lanes 1 and 2). The cell lysate was analyzed ona 4-12% Bis-Tris NuPAGE gel. Lanes 1 and 2 represent the soluble fraction of the cell lysate from the uninduced cells and IPTG-induced cells, respectively. Lane 3 represents the sample collected during stringent washing of the HisTrap column. Lanes 4, 5, and 6 represent the sample collected during the elution step from the HisTrap column, pooled samples after gel filtration, and the last fraction collected during gel filtration, respectively. Samples were analyzed on a 4-12% Bis-Tris NuPAGE gel. Figure S2. Activity analysis of purified full-length NS3. Upon increasing the substrate concentration, no detectable increase in the fluorescence intensity was observed in the case of the full-length His6-NS3, suggesting that the full-length His6-NS3is non-functional in the absence of a fused NS4A cofactor. Figure S3. A standard calibration curve plotted between various concentrations of EDAN [file 12906_2022_3578_MOESM1_ESM.docx]

**Supplementary Materials**

**Journal:** BMC Complementary Medicine and Therapies

**Title:** Hesperidin identified from *Citrus* extracts potently inhibits HCV genotype 3a NS3 protease

Mahim Khan^1^, Waqar Rauf^1^, Fazal-e- Habib^1^, Moazur Rahman^1,2^, Shoaib Iqbal^1^, Aamir Shehzad^1^, Mazhar Iqbal^1^

^1^Health Biotechnology Division, National Institute for Biotechnology and Genetic Engineering College, Pakistan Institute of Engineering and Applied Sciences (NIBGE-C, PIEAS), Faisalabad-38000, Punjab, Pakistan.

^2^School of Biological Sciences, University of the Punjab, Lahore 54810, Punjab, Pakistan

**Corresponding authors:** (i) Mazhar Iqbal, PhD, Professor & Head, Health Biotechnology Division, National Institute for Biotechnology and Genetic Engineering College, Pakistan Institute of Engineering and Applied Sciences (NIBGE-C, PIEAS), Jhang Road, Faisalabad 38000, Punjab, Pakistan. Email: [hamzamgondal@gmail.com](mailto:hamzamgondal@gmail.com).

(ii) Moazur Rahman, PhD, Professor, School of Biological Sciences, University of the Punjab, Lahore 54810, Punjab, Pakistan. Email: moaz.sbs@pu.edu.pk.

**Supplementary Figures:**

**Figure S1:** Cloning strategy, expression and purification of the full-length NS3 of HCV genotype 3a. **(A)** The gene encoding the full-length NS3 was cloned in the pET11a plasmid under the control of the T7 promoter using BamHI and HindIII restriction sites. The construct was used for heterologous expression of the full-length NS3 fused to a polyhistidine (His_6_) tag in *Escherichia coli*. **(B)** Expression analysis of the full-length NS3 in *E. coli* BL21 (DE3) cells (lanes 1 and 2). The cell lysate was analyzed on a 4-12% Bis-Tris NuPAGE gel. Lanes 1 and 2 represent the soluble fraction of the cell lysate from the uninduced cells and IPTG-induced cells, respectively. Lanes 3 to 9 represent samples not related to the current study. The black arrow indicates the band corresponding to the estimated molecular weight (~ 68 kDa) of the full-length NS3. Lane M represents the mobility of proteins with known molecular weights (SeeBlue Pre-stained Protein Marker). (C) Expression analysis of the full-length NS3 in BL21-CodonPlus(DE3)-RIL cells (lanes 1 and 2). The cell lysate was analyzed on a 4-12% Bis-Tris NuPAGE gel. Lanes 1 and 2 represent the soluble fraction of the cell lysate from the uninduced cells and IPTG-induced cells, respectively. Lane 3 represents the sample collected during stringent washing of the HisTrap column. Lanes 4, 5, and 6 represent the sample collected during the elution step from the HisTrap column, pooled samples after gel filtration, and the last fraction collected during gel filtration, respectively. Samples were analyzed on a 4-12% Bis-Tris NuPAGE gel.

**Figure S2:** Activity analysis of purified full-length NS3. Upon increasing the substrate concentration, no detectable increase in the fluorescence intensity was observed in the case of the full-length His_6_-NS3, suggesting that the full-length His_6_-NS3 is non-functional in the absence of a fused NS4A cofactor.

**Figure S3:** A standard calibration curve plotted between various concentrations of EDANS and the generated fluorescence signal. A linear line having an R^2^ value of 0.996 was obtained.

**Figure S4:** Inhibitory effect of the pomegranate pericarp extract (used as a positive control) on the activity of NS3-NS4A. The pomegranate extract significantly inhibited the activity of NS3-NS4A (IC_50_ value of 5.52 ± 0.74 µg/mL) as measured through the validated FRET assay.

**Figure S5:** ESI-MS/MS analysis of *P. granatum* pericarp methanolic extract in negative ion mode.

**Figure S6:** Proposed fragmentation of ellagic acid hexoside based on quasi-ESI-MS^n^ spectra in negative ion mode.

**Figure S7:** Proposed fragmentation of ellagic acid based on quasi-ESI-MS^n^ spectra in negative ion mode.

**Figure S8:** Proposed fragmentation of ellagic acid pentoside based on quasi-ESI-MS^n^ spectra in negative ion mode.

**Figure S9:** Inhibitory effect of ellagic acid on the activity of NS3-NS4A. Ellagic acid significantly inhibited the activity of NS3-NS4A as measured through the FRET assay, yielding an IC_50_ value of 29.62 ± 1.47 µg/mL.

**Figure S10:** ESI-MS/MS analysis of the bitter orange seeds extract in the negative ion mode.

**Figure S11:** Docking of strong (telaprevir) and weak (palmitic acid, linoleic acid, and cerebronic acid) inhibitors in the active site of HCV genotype 3a NS3 protease. The region around the active site selected for docking of compounds is depicted as a blue circle. Active site residues (His57, Asp81, and Ser139) are shown in red, while docked compounds are shown as orange sticks (telaprevir), magenta sticks (palmitic acid), green sticks (linoleic acid), and blue sticks (cerebronic acid). Hydrogen bonds are depicted as blue dotted lines. The interaction of telaprevir, palmitic acid, linoleic acid, and cerebronic acid with active site residues is presented in **(A)**, **(B)**, **(C)**, and **(D)**, respectively.

**Supplementary Tables:**

Table S1: Inhibition trials of telaprevir against NS3-NS4A protease

| **Telaprevir** | **Activity** | | |  |  |  |
| --- | --- | --- | --- | --- | --- | --- |
| **Inhibitor conc. (nM)** | **Slope trial 1st** | **Slope trial 2nd** | **Slope trial 3rd** | **Average Slope** | **StDev** | **Precision** |
| 0 nM | 3.946 | 2.879 | 2.423 | 3.08 | 0.78 | 0.25 |
| 1.3717 nM | 4.176 | 2.087 | 2.395 | 2.88 | 1.12 | 0.39 |
| 4.1152 nM | 4.77 | 3.604 | 1.309 | 3.22 | 1.76 | 0.54 |
| 12.3456 nM | 3.041 | 1.613 | 2.131 | 2.26 | 0.72 | 0.31 |
| 37.037 nM | 3.796 | 1.828 | 1.004 | 2.20 | 1.43 | 0.64 |
| 111.1111 nM | 1.774 | 1.116 | 0.627 | 1.17 | 0.57 | 0.49 |
| 333.3333 nM | 0.5776 | 0.3406 | 0.163 | 0.36 | 0.20 | 0.57 |
| 1000 nM | 1.246 | 0.081 | 0 | 0.44 | 0.69 | 1.57 |

Table S2: Calculation of Linearity, LOD and LOQ using standard curve

| **Linear Equation** | **R^2^ value** | **LOD [3.3*(σ/s)]** | **LOQ**  **[10*(*σ*/s)]** |
| --- | --- | --- | --- |
| y = 7593.8x + 856.46 | 0.9963 | 552.2597 | 1673.514 |

Table S3: Comparison of IC_50_ values of commercial inhibitors used in study with IC_50s_ reported in the literature

| **Sr. No.** | **Inhibitor** | **IC_50_ value (µg/mL)**  **[current study]** | **IC_50_ value (µg/mL) [reported in the literature]** | **Accuracy (%)** | **S-Score** | **Reference** |
| --- | --- | --- | --- | --- | --- | --- |
| 1. | Telaprevir | 0.0475 ± 0.0075 | 0.0387 | 122 | -10.7262 | [1] |
| 2. | Danoprevir | 0.0184± 0.0036 | 0.0146 | 126 | -10.5030 | [2] |

Table S4: Inhibition trials of Danoprevir against NS3-NS4A protease

| **Danoprevir** | **Activity** | | |  |  |  |
| --- | --- | --- | --- | --- | --- | --- |
| **Inhibitor conc. (nM)** | **Slope trial 1st** | **Slope trial 2nd** | **Slope trial 3rd** | **Average Slope** | **StDev** | **Precision** |
| 0 nM | 2.204 | 2.639 | 6.543 | 3.79 | 2.38 | 0.62 |
| 1.3717 nM | 1.677 | 2.153 | 9.123 | 4.31 | 4.16 | 0.96 |
| 4.1152 nM | 1.449 | 1.834 | 9.072 | 4.11 | 4.29 | 1.04 |
| 12.3456 nM | 0.991 | 0.979 | 5.934 | 2.63 | 2.85 | 1.08 |
| 37.037 nM | 0.450 | 0.460 | 2.283 | 1.06 | 1.05 | 0.99 |
| 111.1111 nM | 0.196 | 0.174 | 0.757 | 0.37 | 0.33 | 0.87 |
| 333.3333 nM | 0 | 0.039 | 0.003151 | 0.01 | 0.02 | 1.54 |
| 1000 nM | 0 | 0 | 0 | 0 | 0 | 0 |

**Supplementary references:**

1. Federico A, Aitella E, Sgambato D, Savoia A, De Bartolomeis F, Dallio M, et al. Telaprevir may induce adverse cutaneous reactions by a T cell immune-mediated mechanism. Ann Hepatol. 2015;14:420–4.

2. Miao M, Jing X, De Clercq E, Li G. Danoprevir for the treatment of hepatitis C virus infection: design, development, and place in therapy. Drug Des Devel Ther. 2020;14:2759–74.
